# Prevalence of *Lawsonia intracellularis* and characteristics of gut microbiomes of the infected pigs in Shaanxi province, China

**DOI:** 10.3389/fmicb.2026.1834533

**Published:** 2026-05-11

**Authors:** Yu Wang, Haoyu Zhao, Yuetong Sun, Mingkai Mi, Xiaofei Hu, Dongyang Ye, Xinglong Wang, Lina Dou, Juan Wang, Zengqi Yang

**Affiliations:** 1 College of Veterinary Medicine, Northwest A&F University, Xianyang, China; 2 Key Laboratory for Prevention and Control of Major Ruminant Diseases, Ministry of Agriculture and Rural Affairs, Xianyang, China; 3 Duowei Pharmaceutical Group Co., Ltd., Beijing, China

**Keywords:** 16S rRNA sequencing, *Lawsonia intracellularis*, microbiome, pig, porcine proliferative enteropathy

## Abstract

**Objectives:**

*Lawsonia intracellularis* is an obligately intracellular enteric bacterium that infects intestinal epithelial cells and causes porcine proliferative enteropathy (PPE). This study aimed to investigate the epidemiological prevalence of *L. intracellularis* in large-scale pig farms in Shaanxi Province, China, and analyze the differences in fecal microbial communities of growing pigs with natural *L. intracellularis* infection.

**Methods:**

A total of 672 fecal samples and 300 serum samples were collected from five intensive pig farms in Shaanxi during 2022–2023. Quantitative PCR (qPCR) was used to detect *L. intracellularis* in feces and quantify its fecal load, while enzyme-linked immunosorbent assay (ELISA) was employed to detect the seroprevalence of anti-*L. intracellularis* antibodies in serum. 16S rRNA gene sequencing was performed on fecal samples from *L. intracellularis*-positive and -negative growing pigs.

**Results:**

The results showed an overall fecal positive rate of 15.3% (95% CI: 12.7–18.3%) and a serum seroprevalence of 14.7% (95% CI: 11.0–19.3%) for *L. intracellularis* in the surveyed farms, with growing pigs exhibiting the significantly highest positive rate and fecal pathogen load (*p* < 0.05). Tongchuan exhibited a significantly lower fecal positive rate than the other surveyed regions (*p* < 0.05). Fecal microbial diversity analysis revealed that *L. intracellularis*-positive fecal samples exhibited significantly higher bacterial species richness. LEfSe analysis indicated a significant enrichment of *Lactobacillus* in *L. intracellularis*-positive feces relative to negative samples. Network analysis demonstrated a positive correlation between *Desulfovibrionaceae* and *Lachnospiraceae*, and random forest analysis identified *Erysipelotrichaceae_UCG.003* as the critical microbial biomarker for *L. intracellularis* infection.

**Conclusion:**

This study elucidates the epidemiological characteristics of *L. intracellularis* in Shaanxi and its interaction with the porcine gut microbiome, thereby providing a theoretical basis for the precise prevention and control of PPE, as well as for further investigations into the interactions between the gut microbiota and *L. intracellularis*.

## 1 Introduction

The *Lawsonia intracellularis* is widespread in all pig-keeping continents worldwide and the causative agent of porcine proliferative enteropathy (PPE) (Lawson and Gebhart, 2000; Jacobson et al., 2010; Arnold et al., 2019), characterized by hyperplasia of the epithelial cells of intestinal crypts and, consequently, thickening of the intestinal mucosa (Lawson and McOrist, 1993). Clinical signs and affected age groups vary depending on whether the acute or chronic form of the disease occurs (Lawson and Gebhart, 2000; Jacobson et al., 2003). In young adults, the infection leads to an acute haemorrhagic form of the disease. The bacterium also infects pigs subclinically without obvious clinical signs, still resulting in diarrhea, poor feed conversion, and reduced growth performance, worsened feed conversion, reduced growth performance and significant economic losses (Vannucci and Gebhart, 2014). Besides pigs, *L. intracellularis* has been described in many other mammalian and avian species including rabbits, macaques, mice, dogs, rats and ostriches (Lawson and Gebhart, 2000).

The economic impact of PPE could be important, particularly in grow-finish pigs, result in a reduction in daily weight gain of approximately 3 to 19%. Based on case–control and experimental challenge studies, the value of productivity losses caused by PPE in the finishing phase ranged from $5.98 to $17.34 per marketed pig (Holtkamp, 2019). These losses vary from country to country. This may depend on the severity of the disease. It has been documented that the disease has a high prevalence in China (Wu et al., 2014; Xiao et al., 2022). At present, control of *L. intracellularis* infection is often accomplished by either vaccination or antimicrobial treatment (McOrist and Smits, 2007; Holyoake et al., 2009; Karuppannan and Opriessnig, 2018). Antimicrobial therapy is often widely used rather than expensive vaccine to control the disease during PPE outbreaks in China (Alexopoulos et al., 2006; Wattanaphansak et al., 2019). However, the obligate intracellular nature of *L. intracellularis* prohibits the use of standard antimicrobial susceptibility testing methods that are used for other bacteria (Wattanaphansak et al., 2019). Additionally, the misuse or overuse of antibiotics can lead to increasing antibiotic resistance of pathogenic bacteria, which has become one of the greatest threats to public health (Qiao et al., 2018). Due to antibiotics lead to increased antibiotic resistance of pathogenic bacteria, the control of *L. intracellularis* infection poses challenges for producers and veterinarians (Lloyd and Page, 2018).

The pathogenesis of *L. intracellularis* transmits between pigs by the oral-fecal route (Love and Love, 1977). It can survive in the environment for up to 2 weeks in a temperature range of 5–15 °C (Guedes and Gebhart, 2003). Herds which are completely negative in both, serology and direct pathogen detection like quantitative polymerase chain reaction are rare (Arnold et al., 2019; Xiao et al., 2022). However, contact with the pathogen will not always led to clinical disease (McOrist et al., 1993). Shedding of the pathogen concentration correlates with the severity of lesions in histopathology or immunohistochemistry (Burrough et al., 2015). Moreover, *L. intracellularis* can significantly alter the gut microbiome and that the ileum is the preferential site of *L. intracellularis* infection (Borewicz et al., 2015; Vannucci et al., 2019). Therefore, *L. intracellularis* infection may result in microbial dysbiosis and decrease the immune response of the host to pathogens, thereby exposing the host to other enteric infections, such as *Salmonella* (Leite et al., 2018).

In order to obtain the major transmission routes in different stock classes and production systems, and better understand the underlying microbial community interactions occurring with disease. The current study aimed to determine the presence of *L. intracellularis* and seropositivity in intensive pig production herds in Shaanxi province of China during 2022–2023, and investigate the characterization of the microbiome response to natural *L. intracellularis* infection.

## 2 Materials and methods

### 2.1 Herds and animals

The aim of this study was to provide an overview of the prevalence of *L. intracellularis* in intensive pig production herds in Shaanxi province in China. The herds details are described in Table 1. Growth promoters according to China legislation were not used in the herds and pigs sampled were not subjected to any medication. The fecal and blood samples were collected during the period April 2022 to August 2023 and the samples were taken from the pigs without any clinical symptoms. The fecal samples from pigs corresponded to approximately 5% of all age categories pigs present in the herd at the sampling occasion.

**Table 1:** The detailed of samples information.

Regions	Herd size (sow)	Production type	Age categories of sampled animals	Number of diagnostic materials
Luochuan (109°42′N, 35°78′E)	1,000	Breeding farms	NP, GP, FP, GS, LS, GT, BR	Faeces: 83 Blood: 60
Tongchuan (109°09′N, 35°12′E)	3,300	Two-site farms	NP, GP, FP, GS, LS, GT, BR	Faeces: 223 Blood: 60
Weinan (109°60′N, 34°96′E)	600	Single-site farms	NP, GP, FP, GS, LS, GT, BR	Faeces: 113 Blood: 60
Zhouzhi (108°21′N, 34°17′E)	400	Single-site farms	NP, GP, FP, GS, LS, GT, BR	Faeces: 149 Blood: 60
Chenggu (107°34′N, 33°16′E)	1,200	Breeding farms	NP, GP, FP, GS, LS, GT, BR	Faeces: 104 Blood: 60

Nursery pigs (NP) = 10–25 kg, Growing pigs (GP) = 25–40 kg, Finishing pigs = 40 to till slaughter, Gestation sows (GS), Lactating sows (LS), Gilt (GT) = 50–100 kg, Boars (BR).

### 2.2 Sample collection

Before sampling took place, fecal containers and blood tubes were labelled with the sampling date, sampling material, regions and individual number of the sample which included the age category. Animals were divided into the following seven categories based on their age and weight: nursery pigs (NP) with a bodyweight of approximately 10–25 kg; growing pigs (GP) with a bodyweight of approximately 25–40 kg and finishing pigs (FP) with a body weight of approximately 40 kg to till slaughter; gestation sows (GS); lactating sows (LS); gilt (GT) with a bodyweight of approximately 50–100 kg; boars (BR). During a random walk, a random selection of animals within these seven categories was performed. At least 2 g of native faeces from the rectum of animals per age category and herd were sampled. To avoid cross contamination between the samples, non-sterile gloves had to be worn and changed after each animal. At least 6 mL of blood was collected from the Vena jugularis externa. Considering sample size calculations and economic constraints, blood samples were collected from 10 animals within each age category. The blood samples and fecal samples were derived from different pigs. The boars did not take blood due to biosecurity concerns. Sampled blood was stored overnight to clot at approximately 23 °C and was then centrifuged for 10 min at 2500 g. Subsequently the supernatant serum samples were stored at − 20 °C until they were shipped together with the cooled or frozen fecal samples in refrigerated containers to our laboratory and stored at a temperature of −80 °C until further analysis.

### 2.3 DNA extraction, qPCR and ELISA

A total of 672 fresh fecal samples were collected directly from the rectum of each animal at all age categories. DNA was extracted from 200 mg of each sample using the OMEGA E.Z.N.A.@ Stool DNA Kit (OMEG Bio-tek, Inc. Georgia, USA) following the manufacturer’s protocol. DNA quantity and 260/280 ratios were assessed by Nanodrop. The DNA products was stored at −80 °C. Quantitative real-time PCR (qPCR) of fecal samples was conducted at Northwest A&F University Veterinary Diagnostic Laboratory. The qPCR conditions were as described previously (Opriessnig et al., 2019). The region (nucleotide 583–680) of the 16S rDNA gene of *L. intracellularis* (GenBank acces-sion no. L15739) was monitored with the forward primer, 5′-GCGCGCGTAGGTGGTTATAT-3′; reverse primer, 5′-GCCACCCTCTCCGATACTCA-3′; probe, 5′-FAM-CACCGCTTAACGGTGGAACAGCCTT-TAMRA-3′ (Lindecrona et al., 2002). Additionally, the region of the 16S rDNA gene of *L. intracellularis* (GenBank accession no. L15739) was synthesized and cloned onto the pMD-19T vector to generate the standard plasmid. DNA copy numbers were calculated using the formula: Ct = −3.166 × lg (DNA copy number) + 43.147 (R2 = 0.9991 and efficiency = 106.95%).

Serum samples were assayed by ELISA for the presence of *L. intracellularis* antibodies using a validated, commercially available test kit (Svanovir *L. intracellularis*/Ileitis-AB, Boehringer Ingelheim Svanova) according to the manufacturer’s instructions. Samples with a percentage inhibition (PI) ≥ 30% were classified as seropositive, as specified by the manufacturer.

### 2.4 16S rRNA gene sequencing

DNA extracted from the feces of growing pigs was selected for fecal microbial diversity analysis. Fecal samples from 10 *L. intracellularis*-positive growing pigs (LI-pos group, *n* = 10) and 10 *L. intracellularis*-negative growing pigs (LI-neg group, *n* = 10) were selected for 16S rRNA gene sequencing. The V3-V4 region of 16S rRNA gene was amplified with bar-code-indexed universal bacterial primers (forward primer 338F, 5′-ACTCCTACGGGAGGCAGCA-3′ and reverse primer 806R, 5′-GGAC-TACHVGGGTWTCTAAT-3′) (Yang et al., 2020). The PCR reaction was set up in a 50-μL reaction volume which included 25 μL of 2x Premix Taq (Takara Biotechnology, Dalian Co. Ltd., China), 1 μL of each primer, 50 ng of DNA, nuclease-free water. The PCR cycling parameters included 5 min at 94 °C for initialization; 30 cycles of 30 s denaturation at 94 °C, 30 s annealing at 52 °C, and 30 s extension at 72 °C; followed by 10 min final elongation at 72 °C. The PCR instrument was BioRad S1000 (Bio-Rad Laboratory, CA, US). The PCR products were purified with SanPrep DNA Gel Extraction Kit (Sangon Biotech, Shanghai, China) and quantified by electrophoresis on a 1.5% agarose gel. Sequencing libraries were generated using NEBNext® Ultra™ II DNA Library Prep Kit for Illumi-na® (New England Biolabs, MA, USA) following manufacturer’s recommendations and index codes were added. The library quality was assessed on the Qubit@ 2.0 Fluorometer (Thermo Fisher Scientific, MA, USA). At last, the library was sequenced on an Illumina Nova6000 platform and 250 bp paired-end reads were generated (Guangdong Magigene Biotechnology Co., Ltd. Guangzhou, China).

### 2.5 Sequence analysis

Paired sequences were pre-processed (including assembling paired-end reads, trimming primer, and quality. filtering) using the USEARCH (V10, http://www.drive5.com/usearch/). Sequence analysis was performed by Quantitative Insights Into Microbial Ecology2 (QIIME2) software (Bolyen et al., 2019). By which, representative sequences were clustered into operational taxonomic units (OTUs) at 97% sequence identity using the UPARSE algorithm within USEARCH. The Mothur algorithm was used to compare the representative sequence of each OUT with the non-redundant SILVA database (Quast et al., 2013). All statistical analyses were implemented in R (v.5.1.3). Alpha diversity (i.e., observed richness, Shannon diversity, and evenness diversity) was calculated using the Microbiome package. The similarity among the microbial communities in different samples was determined by principal coordinate analysis (PCoA) and non-metric multidimensional scaling analysis (NMDS) based on Bray–Curtis dissimilarity using Vegan package. Group separation was statistically assessed using permutational multivariate analysis of variance (PERMANOVA; adonis2 function, 999 permutations). The relative abundance of microbiota was examined at different taxonomic levels, and linear discriminant analysis (LDA) effect size (LEfSe) analysis was performed to show the differences between groups, using a Kruskal-Wallis alpha of 0.05, a pairwise Wilcoxon alpha of 0.05, and an LDA score threshold of 3.5. Network analysis was performed based on Spearman’s correlation coefficient (|rho| > 0.8, FDR-corrected *p* < 0.05) as described previously (Matchado et al., 2021), and the networks were visualized using Cytoscape 3.4.0 (Shannon et al., 2003). Random forest analysis was performed using the random Forest package (v4.7-1) in R, based on the OTU relative abundance table. We performed validation using a single training set for estimation in cross-validation. The top 500 most variable OTU features were selected as model input based on variance filtering. Feature importance was evaluated using Mean Decrease Gini and Mean Decrease Accuracy metrics.

### 2.6 Statistical analysis

The pig was defined as positive if genome equivalents specific for *L. intracellularis* or antibodies were detected by qPCR and ELISA, respectively. Data were collected in a spreadsheet program (Microsoft® Excel® Office Professional Plus 2023) and then transferred to a statistic program (SPSS 27.0). The qPCR and ELISA data were performed by chi-square test (SPSS 27.0). Multiple group comparative analysis of microbial diversity was conducted using the Kruskal-Wallis rank sum test, followed by false discovery rate (FDR) correction for multiple comparisons and the Tukey–Kramer *post-hoc* test. The concentration of *L. intracellularis* in feces was analyzed using the Kruskal-Wallis test, followed by Dunn’s post-hoc test with Bonferroni correction for pairwise comparisons. Statistical significance and a tendency towards difference were considered as *p* < 0.05 and *p* < 0.10, respectively.

## 3 Results

### 3.1 Fecal samples: qPCR

To investigate the prevalence of *L. intracellularis* in intensive pig farms in Shaanxi Province, China, we collected 672 fecal samples from clinically healthy pigs for bacterial DNA detection (Supplementary Table 1). The results revealed that 15.3% (95% CI: 12.7–18.3%) of the samples were positive for *L. intracellularis*. As presented in Table 2, the detection rate was the lowest in Tongchuan (7.6%), followed by Luochuan (14.5%); Weinan exhibited the highest detection rate (20.4%), succeeded by Chenggu and Zhouzhi with 20.2 and 20.1%, respectively. Chi-square test analysis revealed a statistically significant difference (*p* < 0.05) in the positive rate of *L. intracellularis* among intensive pig farms across different regions. The number of positive samples in Tongchuan was significantly lower (*p* < 0.05) than other surveyed regions.

**Table 2:** Prevalence of *L. intracellularis* in different regions and growth stages of pigs detected by qPCR.

Regions	Sample quantity	Positive quantity	Positive rates (95% CI)
Luochuan	83	12	14.5 (8.0–24.3)^a, b^
Tongchuan	223	17	7.6 (4.6–12.1)^b^
Weinan	113	23	20.4 (13.6–29.2)^a^
Zhouzhi	149	30	20.1 (14.2–27.7)^a^
Chenggu	104	21	20.2 (13.2–29.4)^a^
Sub-total	672	103	15.3 (12.7–18.3)
Growth stages			
NP	110	9	8.2 (4.0–15.4)^a, b^
GP	141	47	33.3 (25.8–41.8)^c^
FP	93	18	19.4 (12.2–29.1)^b, c^
GS	159	10	6.3 (3.2–11.6)^a^
LS	74	3	4.1 (1.1–12.2)^a, b^
GT	83	15	18.1 (10.8–28.4)^a, b, c^
BR	12	1	8.3 (0.4–40.2)^a, b, c^
Sub-total	672	103	15.3 (12.7–18.3)

Nursery pigs (NP) = 10–25 kg, Growing pigs (GP) = 25–40 kg, Finishing pigs = 40 to till slaughter, Gestation sows (GS), Lactating sows (LS), Gilt (GT) = 50–100 kg, Boars (BR). Superscript letters (a, b, c) indicate statistical differences among groups; groups sharing the same letter show no significant difference (*p* > 0.05).

When stratified by pig growth stages, the detection rate of *L. intracellularis* was the highest in growing pigs (33.3%), followed by finishing pigs (19.4%) and replacement gilts (18.1%). In contrast, lactating sows had the lowest detection rate (4.1%), with pregnant sows (6.3%), nursery pigs (8.2%), and boars (8.3%) showing sequentially higher rates in Table 2. Chi-square and Fisher’s test confirmed a significant difference in positive rates among different growth stages (*p* < 0.05). The number of positive samples in growing pigs was significantly higher (*p* < 0.05) than that in nursery pigs, gestation sows and lactating sows.

### 3.2 Concentration of *L. intracellularis* in fecal samples

**Figure 1:**
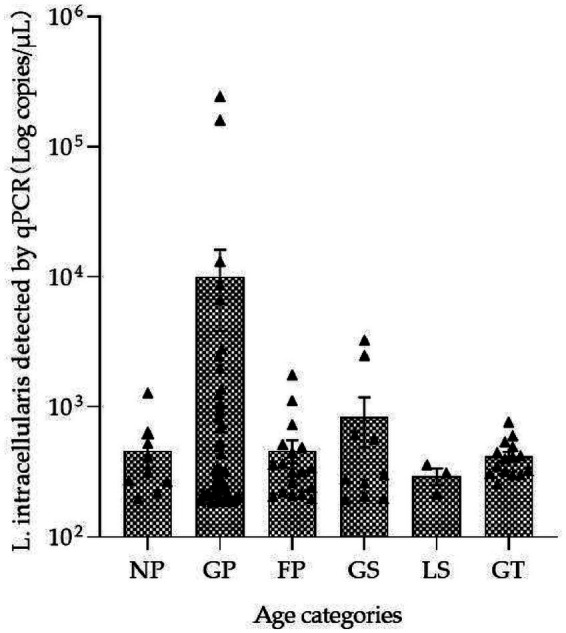
*Lawsonia intracellularis* concentration by pig growth stage. Nursery pigs (NP) = 10–25 kg, growing pigs (GP) = 25–40 kg, finishing pigs = 40 kg till slaughter, gestation sows (GS), lactating sows (LS), gilt (GT) = 50–100 kg.

No significant difference was observed in the fecal concentration of *L. intracellularis* across different pig growth stages (Supplementary Table 2). However, the fecal concentration of this path-ogen in growing pigs was higher than that in other growth stages. Specifically, concentrations of 10⁵ copies/μL were detected in two growing pig samples, whereas this level of concentration was not detected in samples from other growth stages (Figure 1).

### 3.3 Seroprevalence of *L. intracellularis* antibodies

Blood sampling was not performed on boars due to considerations regarding their biosecurity (Supplementary Table 3). The average percentage of samples with antibodies against *L. intracellularis* within herds was 14.7% (95% CI: 11.0–19.3%) (Table 3). There is no significant difference in the serum positive rates of *L. intracellularis* across the five regions. In contrast, the number of positive samples in growing pigs was significantly higher (*p* < 0.05) than that in nursery pigs, gestation sows and lactating sows.

**Table 3:** Seroprevalence of *L. intracellularis* in commercial pigs across different regions and growth stages.

Regions	Sample quantity	Positive quantity	Positive rates (95% CI)
Luochuan	60	6	10 (4.1–21.2)
Tongchuan	60	4	6.7 (2.2–17.0)
Weinan	60	9	15.0 (7.5–27.1)
Zhouzhi	60	12	20.0 (11.2–32.7)
Chenggu	60	13	21.7 (12.5–34.5)
Sub-total	300	44	14.7 (11.0–19.3)
Growth stages			
NP	50	5	10.0 (3.7–22.6)^a, b^
GP	50	13	26.0 (15.1–40.6)^c^
FP	50	9	18.0 (9.1–31.9)^a, b, c^
GS	50	4	8.0 (2.6–20.1)^a, b^
LS	50	3	6.0 (1.6–17.5)^b^
GT	50	10	20.0 (10.5–34.1)^a, c^
Sub-total	300	44	14.7 (11.0–19.3)

Nursery pigs (NP) = 10–25 kg, Growing pigs (GP) = 25–40 kg, Finishing pigs = 40 to till slaughter, Gestation sows (GS), Lactating sows (LS), Gilt (GT) = 50–100 kg, Boars (BR). Superscript letters (a, b, c) indicate statistical differences among groups; groups sharing the same letter show no significant difference (*p* > 0.05).

### 3.4 16S rRNA sequencing analysis

After quality filtering, we obtained 2,513,091 readings with an average length of 432 bp for all samples, 96% of which had a quality score of Q30 and above (Supplementary Table 4). 4,722 operational taxonomic units (OTUs) were obtained through clustering with a 97% identity threshold. The representative sequence of each OTU was aligned against the SILVA 16S rRNA database using USEARCH-SINTAX/BLAST with a confidence threshold of 0.8, resulting in the identification of 24 Phylum, 31Class, 164 Genus and 153 Species.

**Figure 2:**
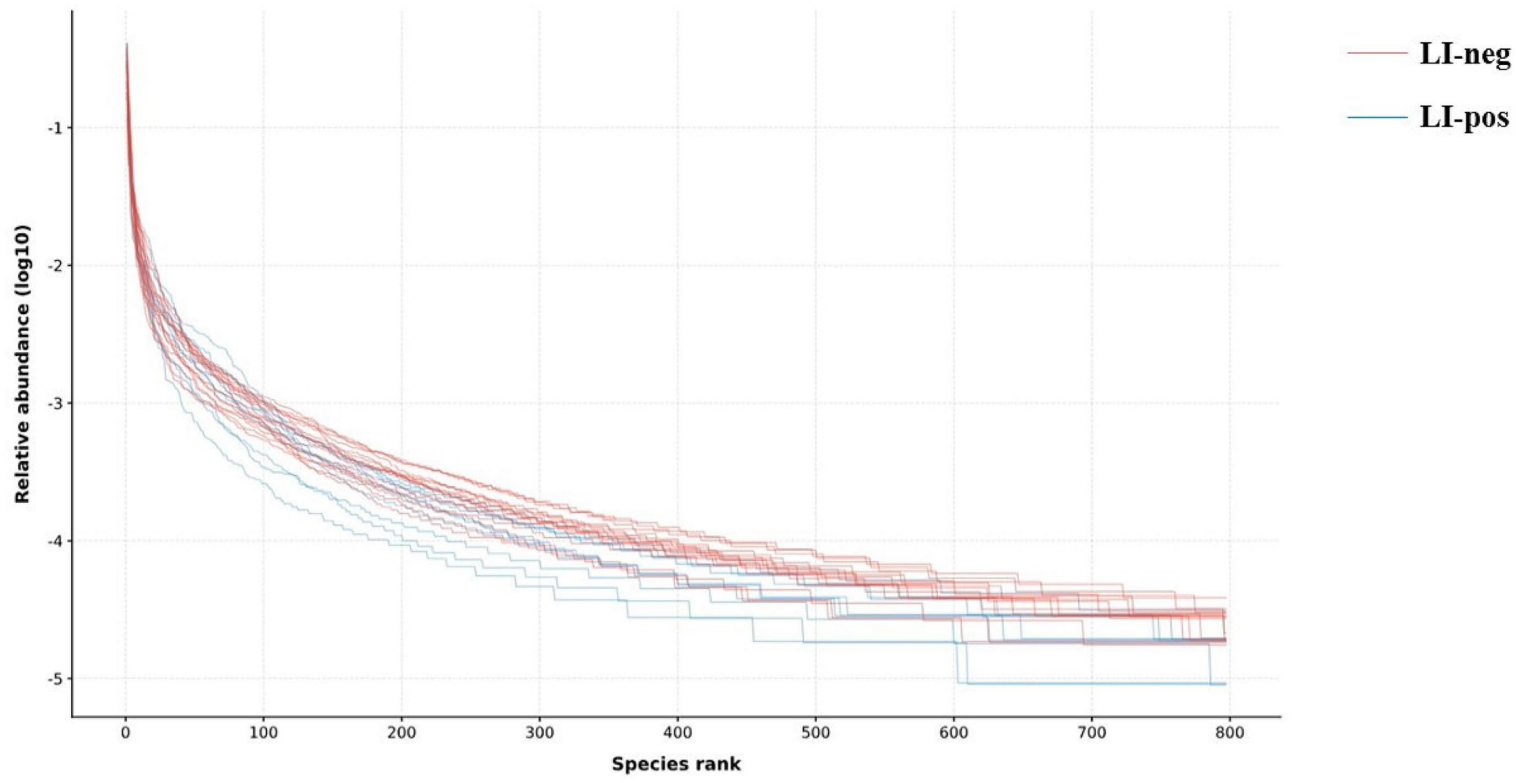
The rank-abundance curves of the *L. intracellularis*-negative group (LI-neg; feces without *L. intracellularis*) and *L. intracellularis*-positive group (LI-pos; feces with *L. intracellularis*).

The rank-abundance analysis visually reflects species richness through the horizontal span of the curve, while the evenness of species composition is indicated by the curve’s shape. As shown in the rank-abundance curves (Figure 2), species evenness appears higher in *L. intracellularis*-negative feces (LI-neg) than in positive ones (LI-pos), as indicated by the flatter curve shape in the LI-neg group. Note that this visual observation is consistent with the trend seen in the rank-abundance curves; however, no statistically significant difference was detected in Shannon or Simpson diversity indices between groups (see Section 3.5.1). Both groups’ rank-abundance curves exhibited the same horizontal width, suggesting comparable species richness between the two groups.

### 3.5 Bacterial community richness and diversity

#### 3.5.1 Bacterial community α-diversity

**Figure 3:**
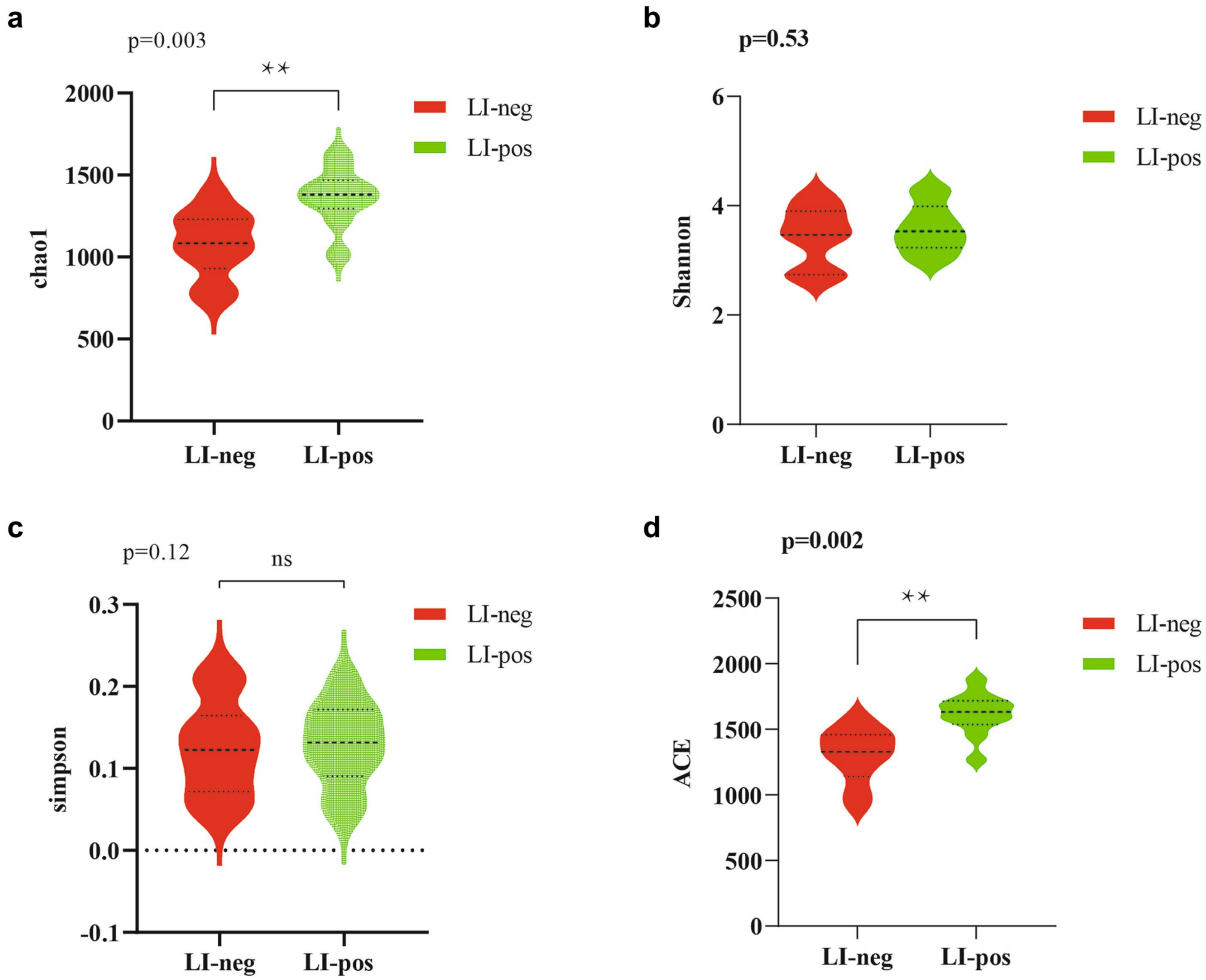
Alpha diversity indices of bacterial communities in fecal samples from *L. intracellularis*-negative (LI-neg, *n* = 10) and *L. intracellularis*-positive (LI-pos, *n* = 10) growing pigs: Chao 1 index **(a)**; Shannon index **(b)**; Simpson index **(c)**; ACE index **(d)**. Statistical comparisons were performed using the Kruskal–Wallis test followed by FDR correction. ^**^p < 0.01; ns, not significant.

The α-diversity indices at four taxonomic levels were calculated to examine differences in feces microbiome richness and diversity across groups (Figure 3). The Chao1 and Abundance-based Coverage Estimator (ACE) indices indicated that the bacterial community abundance and diversity in feces without *L. intracellularis* (LI-neg) were lower than those in feces with *L. intracellularis* (LI-pos) (*p* < 0.01), while there was no difference in the diversity indices Shannon and Simpson (Supplementary Table 5).

#### 3.5.2 Bacterial community β-diversity

**Figure 4:**
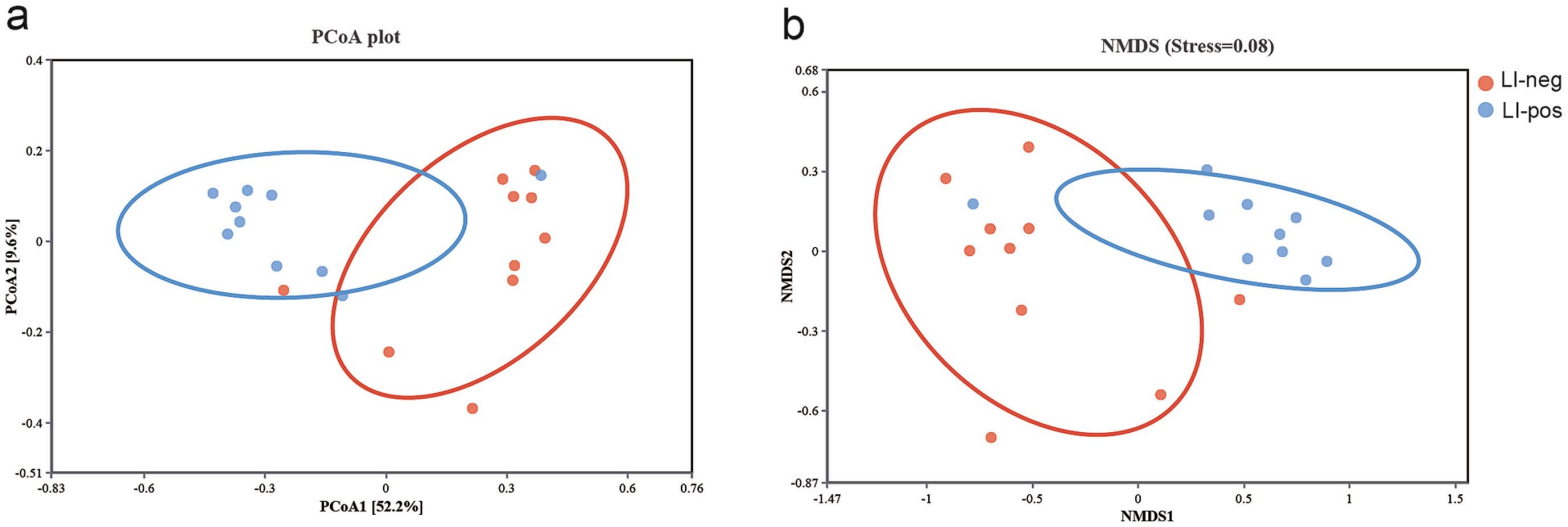
Beta-diversity analysis of fecal microbial communities in LI-neg and LI-pos growing pigs (*n* = 10 per group). **(a)** Principal coordinate analysis (PCoA) based on Bray–Curtis dissimilarity. **(b)** Non-metric multidimensional scaling (NMDS) based on Bray–Curtis dissimilarity. Group separation was assessed by PERMANOVA (999 permutations); *p* < 0.05.

To determine the differences of microbial communities, we performed principal coordinate analysis (PCoA) and nonmetric multidimensional scaling (NMDS) based on Bray-Curtis distance (Figure 4). The fecal microbiota from the LI-neg and LI-pos groups were divided into distinct clusters in both PCoA and NMDS (*p* < 0.05). These results indicate significant changes in the microbial community composition between feces without *L. intracellularis* and those with *L. intracellularis*.

### 3.6 Community composition analysis

**Figure 5:**
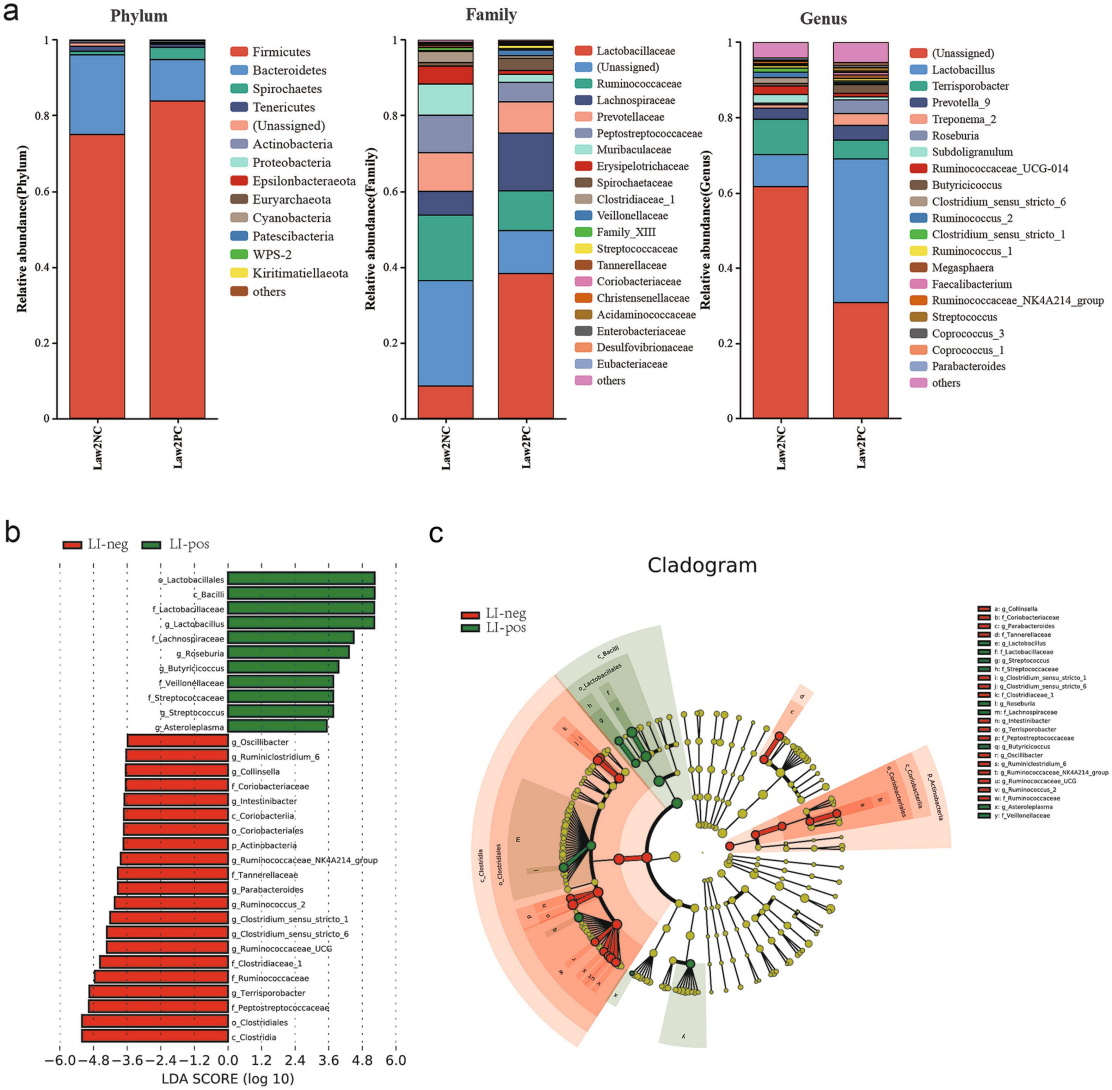
**(a)** Relative abundance of the microbial community at the phylum (left), family (middle), and genus (right). **(b)** Histogram plots of enriched taxa based on linear discriminant analysis effect size (LEfSe) analysis revealed significant in the microbial community between groups. Bacterial taxa with liner discriminant analysis (LDA) score >3.5 were selected as biomarker taxa. **(c)** Cladogram reveals the phylogenetic distribution (p: phylum, c: class level, o: order level, f: family, g: genus level, s: species level).

At the phylum level, *Firmicutes* were the most abundant in all groups, followed by *Bacteroidetes* (Figure 5a), together accounting for more than 90% of the total sequences. Of these predominant phyla, the abundance of *Proteobacteria* was significantly higher in the LI-pos group than in the LI-neg group (*p* < 0.05). At the Family level, the abundance of *Lactobacillaceae*, *Lachnospiraceae*, *Veillonellaceae*, *Streptococcaceae*, *Desulfovibrionaceae* and *Peptococcaceae* was significantly higher (*p* < 0.05) in the LI-pos group compared with the LI-neg group. At the genus level, 26 differentially abundant bacterial genera were identified, and LEfSe analysis revealed that their relative abundance was significantly higher in the LI-pos group compared with the LI-neg group (*p* < 0.05) (Figure 5b). Additionally, it is worth noting that the abundance of *Lactobacillus* and *Erysipelotrichaceae_UCG.003* was significantly higher in the LI-pos group than in the LI-neg group.

### 3.7 Correlation between fecal microbiota in *L. intracellularis*-negative and *L. intracellularis*-positive fecal samples

**Figure 6:**
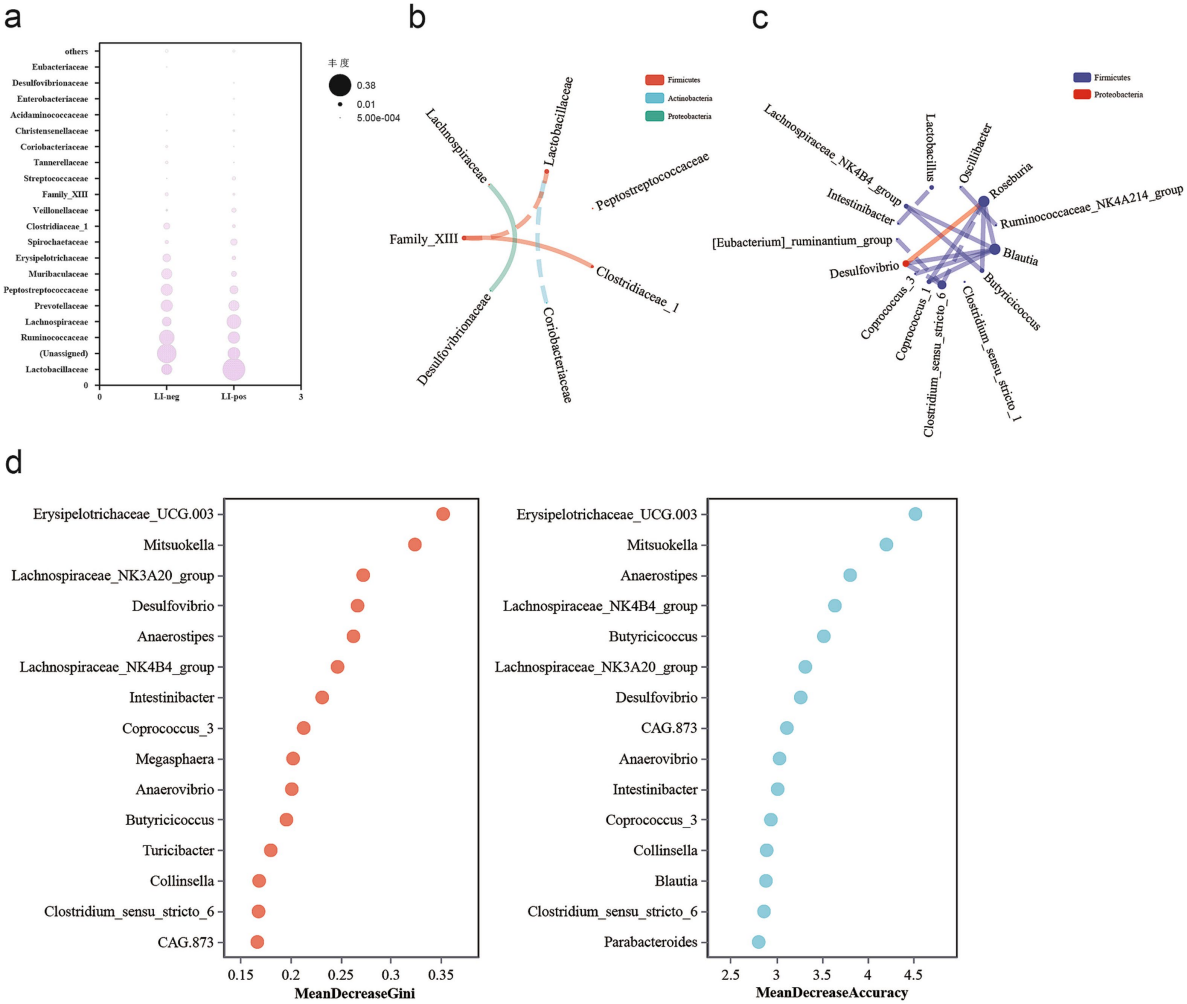
**(a)** Bubble plots at the family level showed the 20 most abundant microbiota by bubble size changes. Spearman network plot of dominant attribute correlations at family **(b)** and genus levels **(c)** (default correlation coefficient ∣rho∣ > 0.8). **(d)** Random forest feature importance analysis identifying the top 15 genus-level bacterial biomarkers discriminating LI-neg from LI-pos fecal samples. We performed validation using a single training set for estimation in cross-validation. Overall classification accuracy: 73.08%; AUC = 68.74% (95% CI: 61.89–75.59%).

Differential microbial taxa detected in the phyla *Proteobacteria* and *Firmicutes*, *Desulfovibrionaceae* and *Clostridiaceae* have a positive correlation with *Lachnospiraceae* and Family XIII (Figure 6b). At the genus level, *Desulfovibrio* and *Roseburia* exhibit a positive correlation (Figure 6c). The bubble plot at the family level also showed that the LI-pos group had the highest abundance of *Desulfovibrionaceae* and *Lachnospiraceae* (Figure 6a). However, in the LI-neg group, the potential pathogenic bacteria belonging to *Erysipelotrichaceae* and *Clostridiaceae* were observed to be more abundant than those in the LI-pos group, while a higher abundance of *Lactobacillus* was detected in the LI-pos group.

The random forest algorithm was employed to identify the microbial species/genes that exert the most significant impact on the target disease (Breiman, 2001). The random forest model achieved an AUC of 0.6874, sensitivity of 88.46%, and specificity of 57.69% in distinguishing LI-pos from LI-neg samples, and Figure 6d presents the top 15 genus-level bacterial features with the strongest predictive power for treatment outcomes. Here, the random forest-based feature importance analysis identified *Erysipelotrichaceae_UCG.003* as the most critical genus-level bacterial biomarker for the predictive model, as it exhibited the highest values in both Mean Decrease Gini and Mean Decrease Accuracy.

## 4 Discussion

With growing public awareness of food safety, inappropriate antibiotic use and residual risks in the breeding industry have become major consumer concerns. Antibiotic consumption in large-scale pig farms in China is substantially higher than that of international counterparts [Organisation for Economic Co-operation and Development (OECD), 2019]. As *L. intracellularis*, the etiological agent of PPE, remains heavily reliant on antibiotic intervention for prevention and control, clarifying the epidemiological characteristics and patterns of this disease during farming is critical to improving the precision of PPE targeted prevention and control. The epidemiological characteristics in this study, a total of 672 normal fecal samples and 300 serum samples were collected from 5 large-scale pig farms in Shaanxi Province. Combined detection techniques involving probe-based qPCR and ELISA were employed to screen for *L. intracellularis*. Subsequently, 16S rRNA gene-based microbial diversity analysis was performed on fecal samples from the growing-finishing phase that were positive and negative for *L. intracellularis*. The ultimate objective of this study was to elucidate the epidemiological characteristics and transmission patterns of the disease in the breeding process, which is of critical significance for enhancing the precision of prevention and control of PPE.

In this study, we analyzed fecal samples randomly collected from healthy pigs of different ages across five large-scale pig farms. The results demonstrated that *L. intracellularis* was detected in all five farms, with an overall fecal positive rate of 15.3% (95% CI: 12.7–18.3%). Since the fecal samples in this study were obtained from clinically healthy pigs, the detection rate of *L. intracellularis* was lower than that reported in previous literature (Arnold et al., 2019; Wang et al., 2024). Nevertheless, this finding still indicates a prevalent status of wide-spread infection with *L. intracellularis* in large-scale pig farms in Shaanxi Province, underscoring the need to strengthen the prevention and control of this disease. Regarding regional distribution, the detection rate of *L. intracellularis* in pig farms in Tongchuan was the lowest among all investigated regions, while no significant difference was found when compared with that in Luochuan. The detection rate in Weinan was 20.4% (95% CI:13.6–29.2%), and its positive rate exhibited no significant difference relative to those in the other regions. Tongchuan is situated on the Loess Plateau with a dry climate, and it is hypothesized that the regional disparities in detection rates may be correlated with local environmental factors including ambient temperature and humidity (Qiu et al., 2022). Regarding the detection results across different growth stages of pigs, the detection rate of *L. intracellularis* in the growing pigs stage was 33.3% (95% CI: 25.8–41.8%), showing a significant difference compared with those in the nursery pigs, gestating sows and lactating sows stages. However, no significant difference was found relative to the finishing pigs, gilt and boars stages. This finding is consistent with previous reports that *L. intracellularis* mainly infects pig populations aged 6–20 weeks (McOrist and Gebhart, 2012), indicating that growing pigs constitute the primary susceptible population to this pathogen and thus targeted prevention and control should be prioritized for this group.

In this study, we further employed the absolute quantitative PCR method to determine the concentration of *L. intracellularis* in fecal samples. The results demonstrated that there was no statistically significant difference in fecal pathogen load among pigs at different growth stages. Among these stages, the growing pig stage exhibited the highest fecal bacterial load, with high pathogen concentrations detected in a subset of individuals. Previous research has confirmed that every 1 log₁₀ unit increase in fecal *L. intracellularis* load is associated with a twofold increase in the correlation between pig growth rate and fecal pathogen shedding (Johansen et al., 2013). In view of the detection data, the positive rate in nursery pigs was relatively low, whereas the highest positive rate was observed in growing pigs. This discrepancy may be related to the presence of maternal antibodies in post-weaning piglets and the use of antibiotics in pig farms (Gómez-Osorio et al., 2025). Following infection with *L. intracellularis*, pigs typically commence fecal shedding of the pathogen approximately 1 week post-infection, and the shedding period can persist for up to 12 weeks (Guedes and Gebhart, 2003). Accordingly, it is hypothesized that infection with this pathogen may occur during the nursery phase when maternal antibody levels wane, with persistent pathogen shedding extending into the growing pig stage. Based on the above-mentioned patterns of pathogen shedding and the temporal characteristics of infection, large-scale pig farms should strengthen targeted prevention and control measures against *L. intracellularis* during the late nursery phase.

In this study, the overall seropositivity prevalence of *L. intracellularis* was 14.7% (95% CI:11.0–19.3%). Regarding regional distribution, no significant difference was detected in the seropositivity rates of *L. intracellularis* among the various pig farms. Numerically, however, the seropositivity rate in Tongchuan pig farms was the lowest, which is consistent with the finding that the fecal positive rate of this pathogen was also the lowest in the same farms. Regarding the detection results across different growth stages of pigs, a higher seroprevalence of *L. intracellularis* was found in growing pigs, at 26.0% (95% CI: 15.1–40.6%), compared with nursery pigs, gestating sows and lactating sows. This result is consistent with the fecal detection rates across these growth stages. Humoral immune responses were first detected 2 weeks after pigs were challenged with a pure culture of *L. intracellularis*, and persisted in some pigs for 13 weeks after exposure (Guedes and Gebhart, 2003). Given that the fecal and blood samples were collected from different pigs, this suggests that the true prevalence of *L. intracellularis* in the pig farms is actually higher. The overall seroprevalence was merely 14.7%, which is consistent with the epidemiological data reported by Wu et al. (2014). To a certain extent, the low true seroprevalence of *L. intracellularis* suggests the longterm use of antibiotics in the investigated pig farms (Lee et al., 2001).

Intestinal microbiota is indispensable to host health via competitive inhibition of pathogens and facilitation of immune system development (Jones and Neish, 2021). Few studies have investigated the microbial differences between normal feces and *L. intracellularis*-carrying feces under natural infection conditions so far. In this study, the detection and positive rates of *L. intracellularis* in both fecal and serum samples were the highest in growing pigs. We performed microbial diversity analysis via 16S rRNA sequencing technology on *L. intracellularis* -positive and -negative fecal samples from growing pigs to characterize the differences in fecal microbial diversity. Meanwhile, understanding the interactions among these microorganisms could provide insights into the complex colonization and infection progression mechanisms of this pathogen (Daniel et al., 2023).

Alpha diversity analysis in this study indicated that *L. intracellularis*-positive fecal samples exhibited significantly higher bacterial species richness than negative samples, as reflected by the Chao1 and ACE indices (*p* < 0.01). Notably, no significant difference was observed in the Shannon or Simpson diversity indices, suggesting that the increase in alpha diversity was driven primarily by species richness rather than evenness. These findings are consistent with those reported by Xu et al. (2023). In their study, the alpha diversity of ileal and cecal microbiota was found to exhibit an increasing trend in pigs challenged with *L. intracellularis*, and they speculated that this increase in alpha diversity might be linked to the elevated abundance of *Desulfovibrio*. These observations align with the results of our present study, where we also detected an increased abundance of *Desulfovibrio* in *L. intracellularis*-positive fecal samples. Beta-diversity was measured using the Bray–Curtis dissimilarity, which is calculated based on species abundance information and is one of the commonly used metrics in ecology to reflect differences between communities (Ricotta and Podani, 2017). In this study, considerable differences in the core microbiota were detected between *L. intracellularis*-positive and -negative fecal samples. Our findings are consistent with Guevarra et al. (2021), who reported that *L. intracellularis* significantly alters gut microbiota structure and composition.

*Firmicutes*, *Bacteroidetes*, *Spirochaetes*, and *Tenericutes* are the four dominant phyla in the intestinal microbiota (Tang et al., 2020). Our results revealed that *L. intracellularis*-positive fecal samples exhibited a higher abundance of *Firmicutes* and a lower abundance of *Bacteroidetes* compared to negative samples. This finding is similar to that reported by Leite et al. (2018), who demonstrated a higher abundance of *Firmicutes* in the pig intestinal microbiome following infection with *L. intracellularis*. It is well known that *Firmicutes* contribute to energy resorption (Houtman et al., 2022). In the present study, we observed that *L. intracellularis*-positive fecal samples exhibited higher abundances of *Lactobacillaceae* and *Lachnospiraceae* than negative samples. Both *Lactobacillaceae* and *Lachnospiraceae* are beneficial families belonging to the phylum *Firmicutes*. Studies have demonstrated that *Lactobacillaceae* strains exert protective effects against *Enteropathogenic* bacteria via their aggregation properties (Zawistowska-Rojek et al., 2022), while *Lachnospiraceae* maintain gut health by producing short-chain fatty acids (SCFA) and suppressing inflammation (Huang et al., 2023). We speculate that the enrichment of these bacterial families may reflect the formation of a diverse core intestinal microbiota at the early stage of natural pathogen infection. By generating SCFA metabolites and reinforcing intestinal mucosal barrier integrity, this microbiota resists the colonization and invasion of pathogenic bacteria (Argüello et al., 2019).

At the genus level, a striking finding of this study was that *Lactobacillus* and *Roseburia* exhibited higher abundances in *L. intracellularis*-positive fecal samples relative to negative ones. This observation is inconsistent with previous studies, which reported that the abundance of *Lactobacillus* was significantly decreased following challenge with *L. intracellularis* (Xu et al., 2023). We speculate that the discrepancy may be attributed to differences in the developmental timeline of the pathogen in the gut microbiota and the effect of the age of the sampled pigs (Argüello et al., 2019). As reported in previous studies, the microaerophilic nature of *Lactobacilli* may explain their survival under elevated redox potential. This environment is induced by reactive oxygen species generated by granulocytes infiltrating inflammatory sites, as occurs in severe intestinal inflammation (Drumo et al., 2016). However, the mechanisms by which *L. intracellularis* infection might boost the growth of these taxa remain unknown.

In the network analysis, each node (circle) represents a single species. Node size corresponds to its relative abundance, and different colors indicate distinct taxonomic groups. Lines connecting nodes represent the correlation between the two species, with line thickness reflecting the magnitude of the correlation coefficient. In the present study, species correlation network analyses were performed at both the family and genus levels. We observed a positive correlation between *Desulfovibrionaceae* and *Lachnospiraceae*. At the genus level, a similar positive association was found between *Desulfovibrio* and *Roseburia*. A recent study showed that *Desulfovibrio* was positively correlated with gut microbiota diversity (Chen et al., 2021), which is consistent with our findings. *Desulfovibrio* species are Gram-negative bacteria capable of reducing sulfate to hydrogen sulfide during the anaerobic respiration of organic matter (Pires et al., 2006), and their sulfide production may contribute to intestinal inflammation (Kushkevych et al., 2019). In the present study, we employed the random forest algorithm to identify key microbial biomarkers capable of discriminating *L. intracellularis*-positive from -negative fecal samples, with a higher relative abundance observed in the LI-pos group compared with the LI-neg group. Our findings demonstrated that the enrichment of *Erysipelotrichaceae_UCG.003* in *L. intracellularis*-positive samples is consistent with the findings of Muwonge et al. (2021), who reported a positive correlation between members of this family and *L. intracellularis* load. *Erysipelotrichaceae* has also been documented to correlate with host metabolic disorders and inflammatory diseases (Wu et al., 2021). Therefore, this finding provides certain theoretical support for subsequent studies on the relationship between *Erysipelotrichaceae* and *L. intracellularis*.

Nevertheless, several limitations in the present study need to be acknowledged. First, the strict biosecurity practices implemented for African swine fever (ASF) control in pig farms have hindered the inclusion of a larger number of farms from more regions in this study. Thus, the current study only provides a cross-sectional snapshot, and the findings may not capture the overall epidemiological status of *L. intracellularis* in pig farms across Shaanxi province in China. Second, samples in this study were restricted to farrow-to-finish sow farms, with no samples collected from specialized finishing farms, which limits a full assessment of the prevalence characteristics of *L. intracellularis* in finishing pig farms. Third, the blood and fecal samples were collected from different individual pigs within the same herd. This design prevents direct comparison of fecal shedding and seroconversion at the individual animal level. In addition, potential confounders-including differences in diet composition, antibiotic use history, housing conditions, herd management practices, and coinfections across the five farms-were not systematically controlled for and may have influenced both *L. intracellularis* prevalence and gut microbiome composition. Future longitudinal studies with matched fecal and serum samples from individual animals, and with standardized farm management data, would help address these limitations. Despite of these limitations, this study still provides insights on the prevalence of *L. intracellularis* on Shaanxi province in China. To our knowledge, this is among the first studies conducted in China to characterize differences in fecal microbial communities between *L. intracellularis*-positive and -negative pigs under natural infection conditions in commercial farms, which provides theoretical support for further research on the relationship between intestinal microbiota and *L. intracellularis*.

## 5 Conclusion

Our investigation revealed a high prevalence of *L. intracellularis* among commercial pig farms in Shaanxi Province, China, and the fecal concentration of this bacterium was the highest in growing pigs. We observed that *L. intracellularis*-positive fecal samples exhibited significantly higher bacterial species richness and distinct community composition compared with negative samples, suggesting that natural infection is associated with a restructuring of the gut microbial community. We also identified *Erysipelotrichaceae_UCG.003* as the most critical microbial biomarker to assist in the diagnosis of *L. intracellularis* infection, although further research is warranted.

## Data Availability

The raw sequencing reads were deposited into the NCBI Sequence Read Archive (SRA) database (Accession Number: PRJNA995047).
